# Advances in Acute Coronary Syndromes: Bridging Gaps in Diagnosis and Treatment

**DOI:** 10.3390/jcm13196003

**Published:** 2024-10-09

**Authors:** Giuseppe Andò, Antonio Micari, Francesco Costa

**Affiliations:** 1 Department of Clinical and Experimental Medicine, University of Messina, Azienda Ospedaliera Universitaria Policlinico “Gaetano Martino”, 98124 Messina, Italy; 2 Department of Biomedical and Dental Sciences and of Morphological and Functional Images, University of Messina, Azienda Ospedaliera Universitaria Policlinico “Gaetano Martino”, 98124 Messina, Italy; antonio.micari@unime.it (A.M.); francesco.costa@unime.it (F.C.)

Acute coronary syndromes (ACS) have long posed a formidable challenge to cardiovascular care, despite significant advancements in both understanding and treatment over the last few decades. ACS, which encompasses conditions such as ST-segment elevation myocardial infarction (STEMI) and non-ST-elevation ACS (NSTE-ACS), continues to be a major cause of morbidity and mortality worldwide. The complexity of ACS pathophysiology, characterized by the rupture or erosion of atherosclerotic plaques and subsequent intracoronary thrombosis, demands a multifaceted approach to treatment. The evolution of antithrombotic therapies has been instrumental in improving outcomes, with potent agents now forming the cornerstone of medical management [1]. Simultaneously, percutaneous coronary intervention (PCI) techniques have undergone significant refinement [2], offering safer, more effective revascularization options, which have dramatically improved procedural success rates and long-term prognosis for patients.

However, despite these advances, the acute presentation of coronary artery disease and the long-term complications associated with ACS remain areas where significant gaps in knowledge and practice persist. The real-world application of novel therapies [3], the optimization of invasive strategies, and the personalization of treatment protocols based on individual patient risk factors and comorbidities all require further investigation. In this context, the role of continuously evolving clinical research becomes even more critical. While current guidelines provide essential frameworks for decision-making and quality assurance in daily practice, new diagnostic algorithms, therapeutic innovations, and emerging technologies consistently challenge established concepts. It is with this in mind that we present this Special Issue, which aims to highlight the most recent evidence in the field of ACS, with a focus on diagnostic and therapeutic innovations that hold the promise of shaping future clinical practice. By addressing both the immediate and long-term management of ACS, the studies within this issue offer fresh perspectives on improving patient care and outcomes.

In the first article, Horne et al. [4] evaluate contemporary predictors of major adverse cardiovascular events (MACE) in patients following PCI. Using data from the Cardiovascular Patient-Level Analytical Platform (CLiPPeR), the study highlights key variables such as cardiogenic shock, chronic kidney disease, and STEMI presentation as significant predictors of MACE. Importantly, the study introduces a parsimonious risk prediction model that may aid clinicians in refining post-PCI care processes to further improve patient outcomes.

Another pivotal study focuses on the critical issue of door-to-balloon (DTB) time in STEMI management. Hsiao et al. [5] retrospectively examine how variations in emergency department arrival times affect DTB time, showing that nighttime arrivals significantly delay cath lab activation and subsequent reperfusion. These findings underscore the need for targeted strategies to streamline care processes, particularly during off-hours, to ensure timely intervention and optimal patient outcomes.

In addressing the complex management of cardiogenic shock, Kanic et al. [6] explore the use of GP IIb/IIIa receptor inhibitors (GPI) in mechanically ventilated patients with myocardial infarction. The study highlights the benefits of GPI in improving TIMI flow post-PCI, without increasing bleeding risk, suggesting selective use of these inhibitors in high-risk patients. However, the authors caution that larger, prospective studies are needed to confirm these findings and clarify the role of GPI in modern ACS care.

Left ventricular remodeling (LVR) in patients with STEMI and multivessel disease is another critical area of focus in this Special Issue. Kim et al. [7] investigate the impact of LVR on long-term outcomes, showing that incomplete revascularization is associated with worse prognosis in patients who develop LVR within the first year post-PCI. These findings have important implications for treatment strategies, particularly in the context of deciding between complete and incomplete revascularization approaches.

Finally, a comprehensive review [8] of the 2023 European Society of Cardiology (ESC) guidelines for ACS management highlights key updates, including recommendations for antiplatelet therapy, the use of advanced imaging techniques, and strategies for managing multivessel disease. The review also points to areas where more personalized approaches to care may be warranted, signaling opportunities for future research and innovation in the field.

While the studies presented in this Special Issue showcase significant advancements in the understanding and treatment of ACS, there remain critical areas of uncertainty and opportunities for future research. One of the most pressing challenges moving forward is the need to refine and personalize risk prediction models, which can more accurately stratify patients based on their unique clinical profiles, comorbidities, and risk factors. The development of more sophisticated, data-driven algorithms will be essential for improving clinical decision-making, particularly in high-risk patients, where timely and precise intervention is paramount. Furthermore, there is a growing need to optimize care delivery during off-hours, such as during the nighttime or weekends, where delays in life-saving procedures such as PCI continue to pose problems. Addressing these logistical and systemic challenges will require innovative solutions that integrate new technologies, streamline hospital processes, and ensure consistent standards of care regardless of the time of presentation.

In addition, future research must explore the integration of emerging technologies, such as artificial intelligence (AI) and machine learning (ML), which hold the potential to revolutionize ACS management. These technologies can provide real-time decision support, improve diagnostic accuracy, and offer predictive analytics to anticipate complications or adverse events. By leveraging large datasets and applying advanced computational techniques, AI and ML can enhance the precision of both diagnostics and therapeutics, allowing for more individualized patient care. The potential of these technologies to transform ACS care cannot be overstated, and they represent a key area for future investigation.

Lastly, while significant progress has been made in pharmacological management, particularly with antiplatelet and antithrombotic [9] therapies, there is still a need for ongoing research into more personalized treatment strategies [10]. As the field moves toward more tailored therapeutic approaches, incorporating patient-specific genetic, molecular, and phenotypic data will be crucial in determining optimal treatment protocols. This precision medicine approach will not only improve outcomes but also minimize the risk of adverse events, ensuring safer, more effective treatments for ACS patients.

In conclusion, we believe that the research presented in this Special Issue offers both a comprehensive overview of the current state of ACS management and a vision for the future. We hope that the studies published here will inspire further research, encourage innovation, and ultimately contribute to improving outcomes for patients facing the challenges of ACS. We extend our deepest gratitude to the authors and reviewers who have contributed to this Special Issue and to the wider cardiovascular research community. The ongoing pursuit of knowledge in ACS will undoubtedly lead to further breakthroughs, and we look forward to seeing how this dynamic field continues to evolve.

## References

[1] Alagna G., Mazzone P., Contarini M., Ando G. (2023). Dual Antiplatelet Therapy with Parenteral P2Y(12) Inhibitors: Rationale, Evidence, and Future Directions. J. Cardiovasc. Dev. Dis..

[2] Tarantini G., Fovino L.N., Varbella F., Trabattoni D., Caramanno G., Trani C., De Cesare N., Esposito G., Montorfano M., Musto C. (2023). A large, prospective, multicentre study of left main PCI using a latest-generation zotarolimus-eluting stent: The ROLEX study. EuroIntervention.

[3] Ando G., Costa F. (2020). Double or triple antithrombotic therapy after coronary stenting and atrial fibrillation: A systematic review and meta-analysis of randomized clinical trials. Int. J. Cardiol..

[4] Horne B.D., Atreja N., Venditto J., Wilson T., Muhlestein J.B., St Clair J.R., Knowlton K.U., Khan N.D., Bhalla N., Anderson J.L. (2024). Contemporary Predictors of Major Adverse Cardiovascular Events Following Percutaneous Coronary Intervention: A Nationally Representative US Sample. J. Clin. Med..

[5] Hsiao Y.T., Hung J.F., Zhang S.Q., Yeh Y.N., Tsai M.J. (2023). The Impact of Emergency Department Arrival Time on Door-to-Balloon Time in Patients with ST-Segment Elevation Myocardial Infarction Receiving Primary Percutaneous Coronary Intervention. J. Clin. Med..

[6] Kanic V., Kompara G., Suran D. (2022). GP IIb/IIIa Receptor Inhibitors in Mechanically Ventilated Patients with Cardiogenic Shock due to Myocardial Infarction in the Era of Potent P2Y12 Receptor Antagonists. J. Clin. Med..

[7] Kim M.C., Lim Y., Ahn Y., Ahn J.H., Lee S.H., Hyun D.Y., Cho K.H., Sim D.S., Hong Y.J., Kim J.H. (2022). Incidence, Predictive Factors and Long-Term Clinical Impact of Left Ventricular Remodeling According to the Completeness of Revascularization in Patients with ST-Elevation Myocardial Infarction and Multivessel Disease. J. Clin. Med..

[8] Licordari R., Costa F., Garcia-Ruiz V., Mamas M.A., Marquis-Gravel G., de la Torre Hernandez J.M., Gomez Doblas J.J., Jimenez-Navarro M., Rodriguez-Capitan J., Urbano-Carrillo C. (2024). The Evolving Field of Acute Coronary Syndrome Management: A Critical Appraisal of the 2023 European Society of Cardiology Guidelines for the Management of Acute Coronary Syndrome. J. Clin. Med..

[9] Gargiulo G., Carrara G., Frigoli E., Leonardi S., Vranckx P., Campo G., Varbella F., Calabro P., Zaro T., Bartolini D. (2019). Post-Procedural Bivalirudin Infusion at Full or Low Regimen in Patients with Acute Coronary Syndrome. J. Am. Coll. Cardiol..

[10] Ando G., De Santis G.A., Greco A., Pistelli L., Francaviglia B., Capodanno D., De Caterina R., Capranzano P. (2022). P2Y(12) Inhibitor or Aspirin Following Dual Antiplatelet Therapy After Percutaneous Coronary Intervention: A Network Meta-Analysis. JACC Cardiovasc. Interv..

